# Barriers and supports for uptake of human papillomavirus vaccination in Indigenous people globally: A systematic review

**DOI:** 10.1371/journal.pgph.0001406

**Published:** 2023-01-06

**Authors:** Shannon E. MacDonald, Lisa Kenzie, Angeline Letendre, Lea Bill, Melissa Shea-Budgell, Rita Henderson, Cheryl Barnabe, Juliet R. Guichon, Amy Colquhoun, Heather Ganshorn, Nancy Bedingfield, Paul D. Vandenboogaard, Robert A. Bednarczyk, Sarah Glaze, Gregg Nelson

**Affiliations:** 1 Faculty of Nursing, University of Alberta, Edmonton, Canada; 2 Cancer Prevention and Screening Innovation, Alberta Health Services, Edmonton, Canada; 3 Alberta First Nations Information Governance Centre, Calgary, Canada; 4 Arnie Charbonneau Cancer Institute, University of Calgary, Calgary, Canada; 5 Department of Family Medicine and Community Health Sciences, Cumming School of Medicine, University of Calgary, Calgary, Canada; 6 Department of Medicine and Community Health Sciences, Cumming School of Medicine, University of Calgary, Calgary, Canada; 7 Department of Community Health Sciences and Pediatrics, Cumming School of Medicine, University of Calgary, Calgary, Canada; 8 Performance Reporting, Alberta Health, Edmonton, Canada; 9 Libraries and Cultural Resources, University of Calgary, Calgary, Canada; 10 Rollins School of Public Health, Emory University, Atlanta, Georgia, United States of America; 11 Tom Baker Cancer Centre, University of Calgary, Calgary, Canada; 12 Department of Obstetrics and Gynecology, University of Calgary, Calgary, Canada; University of Canberra, AUSTRALIA

## Abstract

Despite the availability of effective and safe human papillomavirus (HPV) vaccines that reduce the incidence and impact of cervical cancer and other cancers, HPV vaccine coverage rates remain persistently low and the cervical cancer burden disproportionately high among Indigenous people globally. This study aimed to systematically identify, appraise, and summarize the literature on documented barriers and supports to HPV vaccination in Indigenous populations internationally. Forty-three studies were included and an inductive, qualitative, thematic synthesis was applied. We report on 10 barrier themes and 7 support themes to vaccine uptake, and provide a quantitative summary of metrics. Focusing on Indigenous perspectives reported in the literature, we propose recommendations on community-research collaboration, culturally safe intergenerational and gender-equitable community HPV vaccine education, as well as multi-level transparency to ensure informed consent is secured in the context of reciprocal relationships. Although the voices of key informant groups (e.g., HPV-vaccine eligible youth and community Elders) are underrepresented in the literature, the identification of barriers and supports to HPV vaccination in a global Indigenous context might help inform researchers and health policy makers who aim to improve HPV vaccine uptake in Indigenous populations.

## Introduction

Persistent infection with human papillomavirus (HPV) is responsible for the vast majority of cervical cancers, as well as causing oropharyngeal, vulvar, anal, and penile cancers [1, 2]. This fact raises important questions about healthcare access, appropriateness, safety, and efficacy among populations underserved by current disease treatment and screening models. Vaccines that prevent HPV infection demonstrate long-term effectiveness and acceptable safety profiles, and reduce the risk of cancer incidence [3–5]. However, a number of factors specific to the HPV vaccine and the vaccination process influence its acceptability and uptake. Influential factors in the general population are well described in systematic reviews and include knowledge and perceived risk of HPV disease, misinformation about vaccine safety and perceived (though erroneous) potential that the vaccine could induce sexual promiscuity, cost, the logistics of multi-dose vaccine series delivery, and trust in vaccination programs [6–13]. Additional challenges of administering the HPV vaccine in school settings include the information and consent processes, and student anxiety regarding privacy and pain during vaccination [14]. Further, barriers and supports might be context-specific; for example, access to the HPV vaccine might be facilitated or hindered by parents’ cultural or religious beliefs about sexual activity [13, 15]. Given the unique barriers to health service access for Indigenous people worldwide, barriers and supports to the HPV vaccine may also vary for these populations.

Many Indigenous people inherit and retain unique cultural ways of relating that are distinct from those of colonizing societies [16]; and despite cultural, geographic, and genetic diversity, they share a common experience of colonization. Indigenous people are the descendants of those who experienced settler invasion, occupation, and displacement, many of whom are now determined to secure recognition and protection for the rights and identities of their future generations [17], including rights to health and equitable healthcare. While acknowledging the inherent diversity of culturally distinct groups who exist in some 90 countries [17], we respectfully refer to these groups as ‘Indigenous’, unless otherwise specified, to respect their sovereignty over self-determination and self-identification.

The prevalence of HPV infection is significantly higher among Indigenous people than non-Indigenous people [18–20], with women and their families in these communities being disproportionately affected by cervical cancer incidence, illness, and death [21–23]. Despite this disparity, lower rates of HPV vaccination are found in Indigenous populations globally [24, 25], and the barriers and supports to HPV vaccination are not well understood. We therefore undertook a systematic review to identify the documented barriers and supports to HPV vaccination in Indigenous populations worldwide, with a particular focus on vaccine-eligible youth (<18 years of age).

## Methods

### Aims

Enhancing HPV vaccination is an opportunity to reduce cancer risk among Indigenous people. However, it is critical to center Indigenous voices to understand the context in which HPV vaccine barriers exist and to develop and implement supportive programs. Therefore, we sought first to systematically identify barriers to and supports for HPV vaccination in Indigenous people worldwide as identified in the literature, regardless of who reported the information. Our secondary aim was to amplify the barriers and supports to HPV vaccination *as reported by global Indigenous people themselves*, recognizing that those most directly affected are often most contextually-informed on the social and structural determinants of their own circumstances.

### Self-location of our research team and the application of an Indigenous lens

The land where the majority of our research team resides is today known as the western Canadian province of Alberta. It is home to a number of Indigenous people, including First Nations, Métis and Inuit people, who experience historical and ongoing harmful practices of colonialism and assimilation [26]. As a large team with diversity in cultural backgrounds and social locations, we endeavor to identify our research team through the act of self-location. In addition to taking a leading role on certain parts of the work, our First Nations team members have contributed to and advocated for the health of communities through various roles (e.g. registered nurse, traditional knowledge keeper, executive director, board of directors, and research scientist). As we write and work on colonized lands, the majority of our team members identify as White settlers with varying degrees of systemic privilege rooted in histories of exploitation.

The overarching research project in which this review is situated was a partnership project to explore the reasons behind disparities in HPV vaccine uptake in First Nations people in Alberta. The project’s researchers sought to establish culturally safe processes in our collaborative research partnership with First Nations representatives of a number of First Nations cancer prevention initiatives. By building on previous models of engagement, our team benefitted from best practices that recognize an empowered partnership approach, situating First Nations people, with both experiential and traditional knowledge systems, at the center of the work and creating a foundation for research engagement, implementation, and evaluation [27].

This empowered partnership approach began with creation of a First Nations Elder Advisory Council to determine community interest in the project and to begin a dialogue on the unique needs and concerns of First Nations people regarding HPV and cervical cancer [28]. Members of this Council were engaged during subsequent meetings to respond to advances in activities and to inform systematic review interpretation.

### Data collection and analysis

We employed a broad search strategy, described previously in a published protocol [29], to capture literature related to HPV vaccination, as well as other vaccines, among Indigenous populations globally. Findings specific to HPV vaccination are presented here. We included original research documenting barriers to and supports for HPV vaccination in Indigenous populations worldwide (as defined by the International Work Group for Indigenous Affairs) and excluded studies that: (a) did not discuss barriers or supports to any kind of vaccination; and (b) lacked extractable data (i.e., no relevant data were presented in the study results). A health sciences librarian searched the literature in 11 electronic databases, from inception to June 2017, with updates in December 2019 and May 2021 (see S1 Text for the MEDLINE search strategy). To identify original research not reported in peer-reviewed journal articles, we searched an extensive list of grey literature websites and undertook bibliographic reviews of all included peer-reviewed studies within the first search. Identified literature was screened in two stages (titles and abstract, followed by full-text screening) in duplicate by two team members, with conflicts resolved through discussion. Multiple team members initially assessed methodological quality independently and then conferred to develop agreement as a team by using a universal appraisal tool (Quality Assessment Tool for Reviewing Studies with Diverse Designs—QATSDD) [30]. Data for included studies were extracted into an Excel spreadsheet to capture study characteristics, findings, and the presence/absence of active participation of Indigenous people in the study (e.g., as authors, in program design, in advisory capacity, otherwise).

We used descriptive statistics to summarize study characteristics and undertook iterative, inductive thematic synthesis on extractable barriers and supports, using the systematic review synthesis methods for qualitative research [31]. We conducted thematic analysis of all studies using a step-wise, iterative approach. One researcher coded the extracted data line-by-line using open coding to identify basic themes, which were then reviewed and confirmed by two other researchers. Keeping true to the team’s commitment to grounding observations in contextual insights and expertise of Indigenous people, two team members of First Nation background contributed to reorganization of codes and grouped them into key themes and subthemes, reflecting barriers and supports to HPV vaccination among Indigenous people. For every barrier or support identified, we noted whether it was reported by Indigenous or non-Indigenous participants, an important consideration to mitigate potential for barriers potentially reflecting dominant or stigmatizing biases against medically-underserved groups such as Indigenous people.

In our analysis and discussion, we emphasized the barriers and supports reported by Indigenous people, in order to amplify Indigenous voices and perspectives. We also identified studies that included evidence of community-based approaches, collaborative engagement in pilot studies, or explicit consultation with members of a relevant Indigenous community, so as to promote collective, community ownership of the research process and to highlight culturally-appropriate approaches.

## Findings

### Summary of metrics

**Fig 1:**
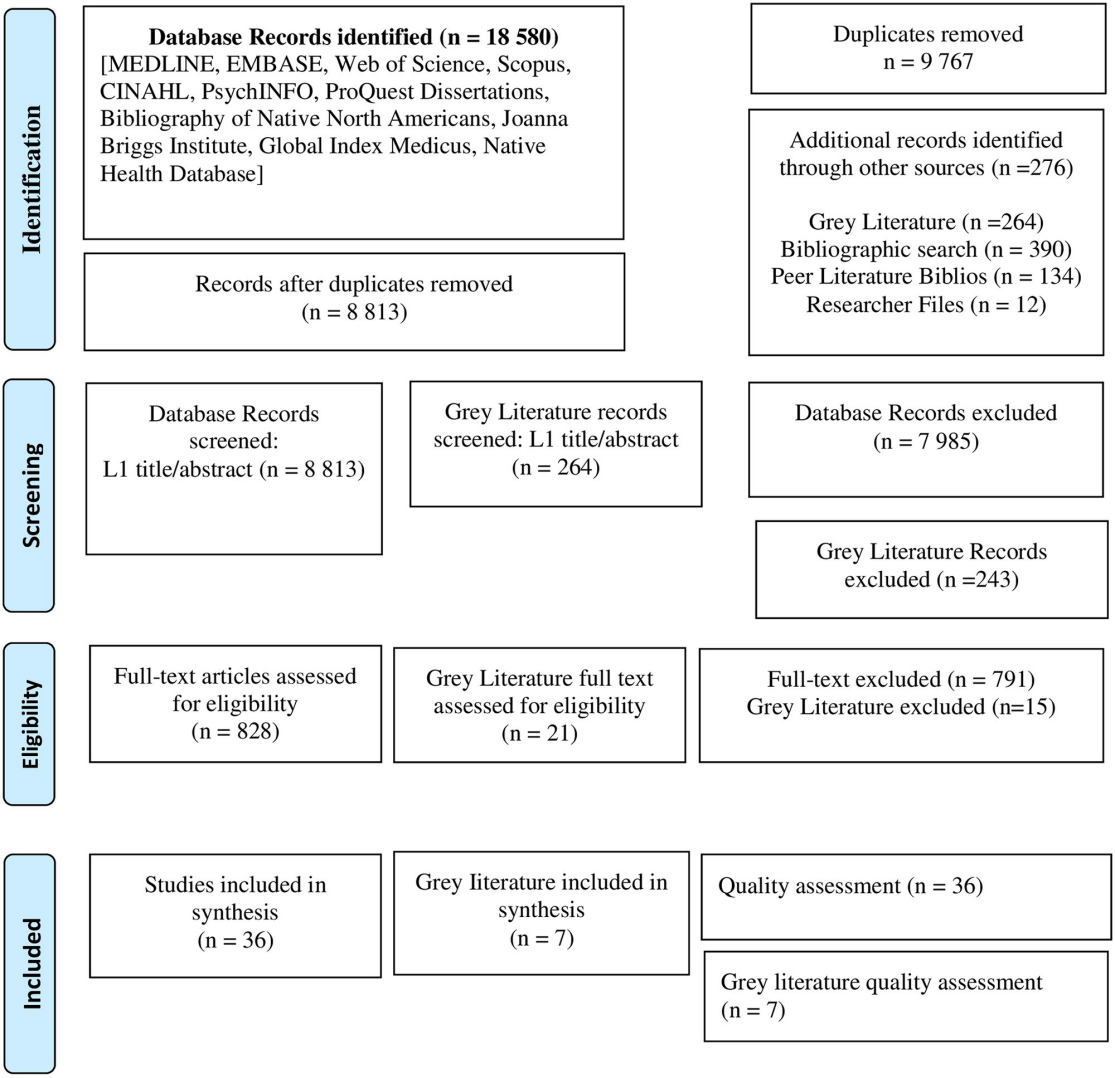
Systematic review PRISMA flow diagram. *From*: Moher D, Liberati A, Tetzlaff J, Altman Dg, The PRISMA Group (2009). *P*referred *R*eporting *I*tems For *S*ystematic Reviews and *M*eta-*A*nalyses: The PRISMA Statement. PLoS Med 6(7): e1000097. doi:10.1371/journal.pmed1000097.

**Fig 2:**
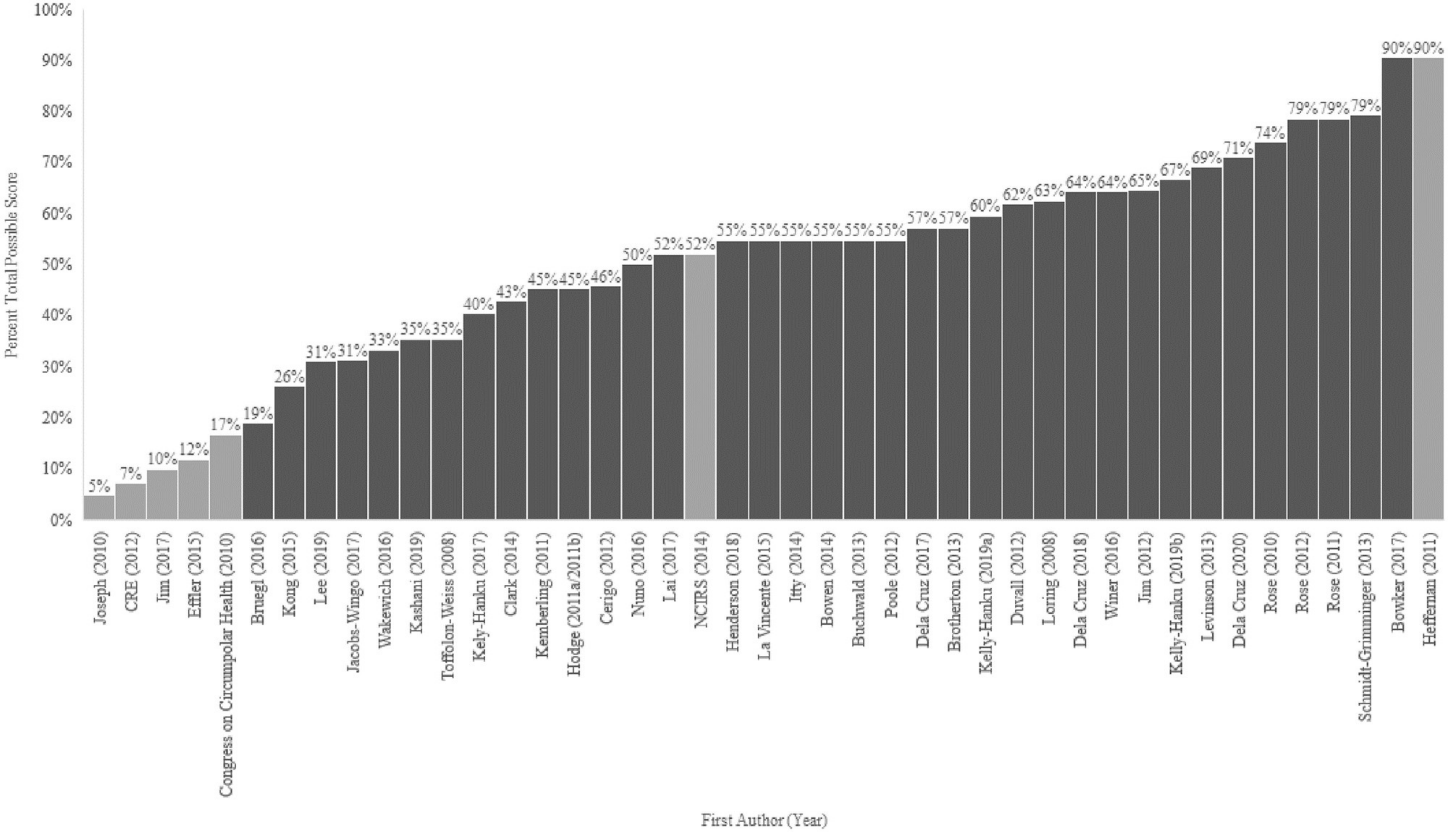
Study quality appraisal histogram (n = 43).

From 16,702 search-generated and de-duplicated records, 43 unique studies (36 original peer-reviewed articles and 7 grey literature sources) met our inclusion criteria (Fig 1); over 95% of these articles and other sources originated from North America and Oceania. Most studies (80%) used qualitative (32%, 14), survey (27%, 12), or mixed methods designs (20%, 9) (Table 1). The majority (58%) involved male and female participants, while a third (38%) involved only females. Participants under 18 years of age were represented in 35% (n = 14) of studies. Of these, only 3 focused specifically on Indigenous youth perspectives—that is, the population itself eligible for the HPV vaccine. Only one of these reported explicit consultation with the Indigenous communities involved. The quality of studies ranged from 5% to 90% of the total possible score quality (Fig 2) [30]. Low scoring studies were retained in the dataset in order to capture the entire breadth of relevant literature, allowing for the inclusion of work for which conventional gold standard health research approaches might not have been possible (e.g. among populations with widespread mistrust, large cultural/geographic diversity, etc.).

**Table 1:** Summary of studies reporting barriers and supports to HPV vaccination in Indigenous people globally.

Author, Year	Study Design	Study Population	Indigenous Subgroups (country)	N (n of Indigenous)	Sex of Target Population^b^	Barrier/Support Reported By^#^	Indigenous Involvement	Indigenous Voices
Dela Cruz et al., 2020	Survey (telephone)	Native Hawaiians, Filipinos, Japanese, and Caucasians	Native Hawaiians (USA)	799 (189)	M, F	parents/caregivers	no	yes
Lee et al., 2019	Survey	White/non-Hispanic, Hispanic/Chicano/Latino, American Indian/Alaska Native; Asian; Pacific Islander/Native Hawaiian; African American	American Indian/Alaska Native (USA)	81 (60)	M, F	community members	no	yes
Kelly-Hanku et al., 2019a	Qualitative (focus groups, semi-structured interviews)	Papuans from Papua New Guinea	Papuans from three provinces: Eastern High-lands Province; Western Highlands Province; and Milne Bay Province (Papua New Guinea)	208 (NR)	M, F	parents/caregivers	no	yes
Kelly-Hanku et al., 2019b	Qualitative (focus groups, interviews)	Papuans from Papua New Guinea	Papuans from three provinces: Eastern High-lands Province; Western Highlands Province; and Milne Bay Province (Papua New Guinea)	82 (82)	M, F	community members	no	yes
Kashani et al., 2019	Mixed methods (Semi-structured interviews)	American Indian/Alaskan Natives and the health care providers who serve them	American Indian/Alaskan Native (USA)	4,722 (4,722) and 14 health care providers (NR)	M, F	healthcare providers	no	no
Dela Cruz et al., 2018	Survey (telephone)	Hawaiians, multicultural	Native Hawaiian (USA)	799 (189)	M, F	parents/caregivers	no	yes
Henderson et al., 2018	Qualitative (sharing circles)	First Nations	First Nations from Cree, Dene/Sarcee, Stoney Nakoda, and Blackfoot cultural backgrounds (Canada)	24 (24)	M, F	community members	yes	yes
Bowker, 2017	Survey	Northern Plains American Indian women	American Indian (USA)	89 (89)	F	community members	yes	no
Jacobs-Wingo et al., 2017	Mixed methods (semi-structured interviews)	American Indians	American Indian (USA)	12,500 (12,500) 35 (NR)	M, F	healthcare providers	no	no
Dela Cruz et al., 2017	Qualitative (face-to-face or telephone interviews)	Hawaiians, multicultural	Native Hawaiian (USA)	20 (5)	F	parents/caregivers	no	yes
Lai et al., 2017	Mixed methods (focus groups, surveys)	Utah multicultural population	American Indian/Alaskan Native, Native Hawaiian/ Pacific Islander (USA)	228 (93)	M, F	parents/caregivers	yes	yes
Kelly-Hanku et al., 2017	Qualitative (semi-structured interviews, focus groups)	Papuans from Papua New Guinea	Papuans from three provinces: Eastern High-lands Province; Western Highlands Province; and Milne Bay Province (Papua New Guinea)	208 (208)	M, F	community members	no	yes
Jim et al., 2017	NR	Males and females aged 9–26 served by Indian Health Services	NR Native American (USA) Winnebago Tribe of Nebraska (USA)	NR (NR) 140 (140) NR (NR)	M, F	study author	no	no
Wakewich et al., 2016	Qualitative (focus groups, interviews)	Canadian First Nations and the health care providers serving them	First Nations, Northwestern Ontario on reserve (Canada)	69 (69) women 16 (12) HCPs	M, F	community members (women) and healthcare providers	yes	yes
Winer et al., 2016	RCT (surveys)	American Indians in Arizona	American Indians, Northeast Arizona on reserve (USA)	97 (97)	F	parents/caregivers	yes	yes
Nuno et al., 2016	Survey (online)	University students	American Indian/Alaskan Native at University of Arizona (USA)	284 (36)	F	vaccine eligible individuals (>18 years)	no	yes
Bruegl et al., 2016	Survey	Members of the Association of American Indian Physicians	American Indian/Alaskan Native (USA)	60 (47)	M, F	healthcare provider	no	yes
La Vincente et al., 2015	Survey (telephone)	Fijians from the Republic of Fiji	Fijians (Republic of the Fiji Islands)	293 (293)	F	parent/caregiver	no	yes
Kong et al., 2015	Retrospective review (charts)	Australian Aboriginals	Aboriginal (Australia)	1,814 (1,814)	M, F	vaccine eligible individuals (both <18 and >18 years)	no	no
Effler, 2015	Retrospective review (database)	Children in the school-based HPV Immunization Program in Western Australia	Aboriginal (Australia)	30,508 (≥1006)	M, F	study author	no	no
Itty et al., 2014	Qualitative (focus groups)	University students	American Indian (USA)	53 (53)	M, F	vaccine eligible individuals (>18 years)	yes	yes
Bowen et al., 2014	Qualitative (focus groups)	Native Americans	Native American (USA)	102 (102)	NR	parents/ caregivers	no	yes
Clark, 2014	Qualitative (semi-structured interviews)	Peruvian indigenous people	Cantagallo and Shipibo-Konibo People (Peru)	30 (30)	F	parents/caregivers	no	yes
National Centre for Immunisation Research & Surveillance (NCIRS), 2014	Retrospective review (registry)	People aged 12–26 in Australia as part of the National Vaccine Program	Indigenous (Australia)	1,463,790 (NR)	M, F	study authors	no	no
Brotherton et al., 2013	Retrospective review (registry)	Australians	Aboriginal and Torres Strait Islanders from Queensland and Northern Territory (Australia)	NR (NR)^a^	F	study authors	yes	no
Schmidt-Grimminger et al., 2013	Mixed methods (tribal focus groups, interviews)	American Indians in South Dakota	Northern Plains American Indian (USA)	73 (73)	F	healthcare providers; vaccine eligible individuals (all ages), parents/caregivers	yes	yes
Buchwald et al., 2013	Survey	Northern Plains women living in South Dakota	American Indian (USA)	352 (194)	F	community members	yes	no
Levinson et al., 2013	Mixed methods	Peruvians living in Manchay, Peru	Peruvian Indigenous People (Peru)	352 (NR)	F	parents/caregivers	yes	yes
Cerigo et al., 2012	Mixed methods (surveys, focus groups)	Canadian Inuit	Inuit (Canada)	175 (175)	F	community members	yes	no
Poole et al., 2012	Retrospective review (registry)	New Zealand children	Maori (New Zealand)	8,665 (772)	F	study authors	no	no
Rose et al., 2012	Survey	New Zealand school children in the Wellington region	Maori (New Zealand)	769 (126)	M, F	parents/caregivers	no	no
Jim et al., 2012	mixed methods (surveys, semi-structured interviews)	Providers in Indian Health Service, Tribal and Urban Indian Healthcare Facilities who work with the AI/AN population	American Indian/Alaskan Native (USA)	268 (268) survey 51 (NR) interview	M, F	healthcare providers	yes	no
Duvall & Buchwald, 2012	Survey	Healthcare providers serving American Indian Tribes in Washington State	American Indian (USA)	NR (31 clinics)	NR	healthcare providers	yes	no
Centre of Research Excellence (CRE), 2012	Qualitative (roundtables and workshops)	Aboriginal health roundtable to identify research priorities	Aboriginal and Torres Strait Islander (Australia)	30 (NR)	M, F	public health/policy leader^c^	no	no
Kemberling et al., 2011	Qualitative (interviews)	Alaska Native adolescents	American Indian/Alaskan Native (USA)	79 (79)	F	vaccine eligible individuals (<18 years)	yes	yes
Rose et al., 2011	Survey	Teachers of Indigenous students in Wellington region of New Zealand	Maori, Pacific Islander (New Zealand)	456 (77)	M, F	teachers and support staff	yes	no
Hodge et al., (2011a & 2011b)	Mixed methods (surveys, focus groups)	University students	American Indian (USA)	57 (57)	M, F	vaccine eligible individuals (<18 years)	no	yes
Heffernan, 2011	Qualitative (semi-structure interviews)	Aboriginal, Anglo or Chinese dependency parents residing in Melbourne	Aboriginal (Australia)	126 (85)	F	parents/caregivers, healthcare providers	yes	yes
Rose et al., 2010	Survey	Parents of children attending school in the Wellington region	Maori, Pacific (New Zealand)	769 (183) 15 schools	M, F	parents/caregivers	yes	yes
Joseph & Menzies, 2010	Survey	Service providers of Aboriginal and Torres Strait Islander Australian females	Aboriginal or Torres Strait Islander (Australia)	NR	F	study authors	no	no
Proceedings of the 14th International Congress on Circumpolar Health, July 11–16, 2009, Yellowknife, Canada, 2010	Qualitative (workshop)	Aboriginal Canadian vaccine recipients	Aboriginal (Canada)	50 (NR)	M, F	study authors	yes	no
Toffolon-Weiss et al., 2008	Qualitative (focus groups)	Alaska Native parents	Alaska Native (USA)	79 (79)	M, F	parents/caregivers	yes	yes
Loring, 2008	Mixed methods (key informant interviews)	New Zealand children	Maori (New Zealand)	8,642 (1,902) 5 (NR)	M, F	public health/policy leader^c^	yes	no

NR = not reported

^#^ Indigenous groups, unless otherwise stated

^a^ 38.8% in the Northern Territory and 5.8% in Queensland self-identified as Indigenous on consent forms; denominator not reported

^b^ Sex of the study target population: M = male, F = female, M,F = both male and female

^c^ Ethnicity unknown

From the 43 included studies, we identified 43 unique barrier codes and 35 unique support codes, which were grouped into 10 barrier themes and 7 support themes (Table 2). We also identified the frequency with which a code appeared in the extracted data and identified these as distinct ‘data points’. Because codes could arise more than once within any given study, the number of data points exceeded the number of studies, themes, or codes. Of 553 total data points identified, there were 365 ‘barrier’ data points from 41 studies and 184 ‘support’ data points from 36 studies. Thirty-two studies reported both barriers and supports, while 8 studies reported only barriers and 3 studies reported only supports.

**Table 2:** Barriers and supports for HPV vaccination in global Indigenous people.

Theme	Code	Data points* reported by all sources in the literature	Data points* reported by Indigenous people in the literature
***Barriers***			
Beliefs	Sex & cultural taboos	26	17
	Risk perception	11	9
	Age-appropriateness	11	7
	Effectiveness-necessity	8	6
	HPV treatment & healing	6	4
	Moral/religious	3	-
	Compulsory vaccine	2	1
Concerns about vaccine safety & side-effects	Non-specific concerns	18	13
	Fear of pain	9	6
	New vaccine with unknown effects	9	7
	Implementation delay	7	5
	Indigenous-specific side-effects	5	5
	Infertility & sterilization	4	4
	Vaccine testing on Indigenous peoples	4	4
	Contracting disease	3	3
	Autism	3	3
	Death	1	1
Lack of effective knowledge dissemination	Knowledge of HPV vaccine	32	25
	Knowledge of HPV disease & outcomes	16	13
Social determinants of health service access	Physical location	12	4
	Individual cost	7	6
	Multi-dose vaccine	7	1
	Health literacy	5	1
	School attendance	4	1
	Program funding	4	-
	Income	4	-
	Indigenous inequities	1	-
Colonial systems and processes	Provider systems	19	4
	Informed consent processes	16	10
	Policy systems	4	1
	Community systems	3	2
	Racist policies	1	-
Multigenerational and social influences	Parent or caregiver	9	6
	Media	8	5
	Stigma	7	5
	Peer	4	4
Lack of healthcare provider recommendation	Vaccine recommendation & education	13	5
	Trust & time	4	3
	Sexual assault	1	1
	Racism	1	1
Distrust	Perceived vaccine intent	5	4
	Distrust in the government	3	3
	Distrust in healthcare workers	3	3
	Distrust in pharmaceutical companies	2	2
	Anti-immunization sentiment	2	1
	Distrust in the medical system	1	-
Lack of culturally-appropriate awareness campaigns	Awareness of HPV vaccine	8	6
	Awareness of HPV disease & outcomes	7	5
Gender- and age-specific disparities in health education delivery	Oral knowledge & sharing stories	8	7
	Gender disparities	5	3
	Culturally-inappropriate delivery	4	1
	Exclusion of elders	1	1
***Supports***			
Community-oriented health education delivery	Community-level discussion	16	13
	Educational support sessions	5	2
	Intergenerational health education	5	5
	Honesty & transparency	5	4
	Vaccine normalization & ownership	5	3
	Indigenous languages	4	2
	Indigenous representation and relevance	3	2
Equity-oriented systems and processes	Provider systems	10	-
	Informed consent processes	7	3
	Policy systems	6	-
	Reminders & follow-up	5	1
	Collaboration with Indigenous leaders	4	1
	Incentives	2	1
Affirmative multigenerational and social influences	Parent or caregiver	9	6
	School	8	3
	Community leaders and elders	5	4
	Peer	5	4
	Media	4	3
Consistent healthcare provider recommendation	Vaccine recommendation & education	11	6
	Trust & time	9	4
	Indigenous status of the provider	4	1
Social and cultural determinants of health	Physical location	5	1
	Vaccine recipient autonomy	5	6
	Gender equity	5	3
	Convenience	5	-
	Early first dose	4	1
	Program funding	3	1
	Previous childhood vaccine	2	2
	Participation in cultural practices	1	-
	Income	1	-
Beliefs	Protection from cancer	7	6
	Vaccine safety	3	1
Increased HPV knowledge	Knowledge of HPV vaccine	6	3
	Knowledge of HPV disease & outcomes	3	1

*Data points = the number of times a code arose in the data; codes may arise more than once in any given study

For each data point we identified who reported each barrier or support, and further classified each voice as belonging to the ‘Indigenous community’ (e.g. vaccine-eligible youth <18 years old, vaccine-eligible young adults ≥18 years old, parents/caregivers, health workers, or general community members), or non-Indigenous ‘colonized systems’ (e.g. authors/academic researchers, healthcare providers, public health/policy leaders, or schoolteachers and educational staff). Indigenous voices were represented through surveys, interviews, focus groups, and sharing circles, with 55% of the data points reflecting perspectives reported among Indigenous populations globally. Barriers and supports were reported by parents/caregivers (15 studies), health workers (4 studies), community members (4 studies), and vaccine-eligible youth (3 studies) and young adults (5 studies). Of 24 studies, six reflected Indigenous voices from more than one ‘reported by’ category. The reported barrier and support themes and codes are outlined in Table 2.

To determine which studies were conducted in a participatory manner through Indigenous community engagement, we incorporated ‘evidence of user involvement in design’ as a measure when using the QATSSD tool for quality appraisal. Indigenous people were, to some degree, engaged and/or involved in 47% of the studies identified in this review (Table 1). However, only 14% of studies reported explicit or formal consultation with, or involvement of, Indigenous groups in any stage of the study process, including planning and implementation.

### Thematic barriers to HPV vaccination

#### Beliefs

Beliefs about sexual behavior were the most commonly reported barrier among those who identified as Indigenous, namely the perception that the vaccine might encourage sexual behavior among young people. These beliefs were particularly evident when concepts of cultural taboo were brought forth. For instance, despite overall support for vaccination in a community in Papua New Guinea, there were concerns expressed by community members about a perceived increase in promiscuity once disease protection was acquired from the HPV vaccine; some participants believed that there might be a community-wide increase in the sex trade [32]. In settings where public health messaging focused on the sexually transmitted nature of HPV, healthcare providers suggested that explicit discussions about sex were needed; such discussions was seen as problematic by Indigenous community members where cultural taboos about discussing matters related to sex, such as sexually transmitted infections (STIs), may persist (e.g., from cultural or historical factors). One schoolteacher in an urban area of Papua New Guinea expressed that discussion of sex or sexual organs in their village was unacceptable [33]. The belief that the HPV vaccine might increase promiscuity was echoed by vaccine-eligible youth in that study. When asked how her community would respond to HPV vaccination messaging focused on STI prevention, one teenage participant said that many would be opposed because of perceived increases in prostitution after vaccination, despite no evidence of any such association [33].

The age appropriateness of the vaccine was a common area of inquiry in surveys about the barriers and supports to HPV vaccination among Indigenous people [34–38]. In one survey among Native Hawaiians, 39% of parents indicated that a reason they did not consent to vaccination of their daughter(s) against HPV was a concern about age (e.g., ‘too young to get the vaccine’) [35]. Within focus groups in the United States, community discussions surrounding the appropriate age for vaccination did not result in clear consensus on preferred vaccine timing for their youth, due in part to the complexities associated with the onset of sexual activity (e.g., general fear that vaccination might somehow excuse sexual initiation) [39]. In Australia, Indigenous community members felt the preferred age for vaccination was largely determined by the physical and emotional readiness of a vaccine-eligible child [40].

There is some evidence that vaccine-eligible Indigenous young adults viewed their risk of contracting an oncogenic strain of HPV as low, which further reduced their vaccine acceptance. In a focus group study of post-secondary students in the southwest United States [41], students voiced opinions that virtually everyone contracts HPV, but not all cases of HPV would necessarily result in cancer; that the HPV vaccine provides minimal protection against all cancer-causing strains of HPV; that vaccination prevents infection from only certain strains of HPV; and that there are alternative prevention options for cervical cancer (namely Pap smears and treat the disease of identified irregularities). These personal risk perceptions played a role in decisions not to obtain the HPV vaccine. Students also discussed traditional approaches to cervical cancer prevention and treatment modalities that exist outside of western biomedical model, including belief in traditional medicines that are customary in their American Indian communities [42].

#### Multigenerational and social influences

Given that the HPV vaccine is offered predominately to youth under the age of 18, parents or caregivers might refuse to consent for their children to be vaccinated in school, or might influence the autonomous decision of their adolescent to seek the vaccine for themselves [41, 43, 44]. Multigenerational caregivers (i.e. including parents and grandparents) might be reluctant to support their daughters/granddaughters in receiving the HPV vaccination, due to the sexual connotations attached to the immunization. One health worker expressed that, in the Northern Plains American Indian community, the father would not consent to vaccination due to misconceptions held about promiscuity [44]. In a different circumstance, three women in Peru reported that their husbands prohibited their daughters from receiving the HPV vaccine [45]. It can be challenging to overcome these barriers because many families are unwilling to discuss sexual behavior. An American Indian university student stated that her family did not openly discuss sexual activity, so conversation about a vaccine that protects someone from HPV would have been dismissed [41].

Negative media reports can increase social stigma and fear of side-effects, and lead to community spread of misinformation regarding the HPV vaccine [40, 44, 46]. Interview participants in Australia indicated a need for public media members to report on the vaccine ‘responsibly’, after a local news broadcast erroneously stated that the HPV vaccine was compulsory [40]. In other communities, some members reported that there had been insufficient information provided through the media to remote communities, leaving Indigenous health workers feeling insufficiently trained to educate community members about the risks and benefits of the vaccine [40].

#### Concerns about vaccine safety and side-effects

During interviews with vaccine-eligible youth in Alaska [47], younger teens reported fear of pain associated with the vaccine, and older teens cited concern regarding side effects. The fear of short- or long-term side effects was reported to have negatively influenced the decision to be vaccinated against HPV. Fear of long-term side effects that might be life threatening was one area of concern, with one Arizona university student stating that something seemed “seriously wrong” with a friend who had just received the vaccine [41].

Concerns about non-specific safety issues and unwanted side effects led Alaska Native parents to delay HPV immunization for their children [48]. Many Indigenous parents in the Northeastern United States did not trust a ‘new’ vaccine, and simply preferred to wait to vaccinate their daughters until more information about the long-term effects were known [39]. Among 44 non-consenting parents in a telephone survey in Fiji, 52.3% cited concerns about HPV vaccine safety as the primary reason for withholding consent. These parents commented that the vaccine was new in their country and that they had heard negative information about the vaccine from friends and in the media [46]. Fears regarding HPV vaccine safety can be so pervasive that even health workers in Indigenous communities can be affected, and some American Indian providers reported apprehension in recommending it [44]. Mothers in Alaska expressed discomfort in consenting to a new vaccine for their children, stating that some side effects might not be known until many children are vaccinated. Another parent was afraid their child might contract the disease as a result of vaccination [48].

Concerns about alleged side-effects unique to Indigenous bodies were raised by community members in various studies. In Victoria, Australia, one parent asked whether the clinical trials had been conducted with Indigenous women, stating “Our immune systems are set for things in the land…if this has not been tested on us, and the vaccine has things that are not from the land, it could make us very sick” [40]. A vaccine-eligible young adult in the United States expressed that there is a lack of understanding about how Indigenous bodies will react to HPV vaccinations [41]. Stories from Australia of women losing their hair after vaccination echoed these concerns about Indigenous-specific side-effects or reactions [40]. Other perceived risks, such as fear of autism, were cited as reasons to be cautious in decision-making [41]. Indigenous parents in the United States perceived all these risks for their daughters to be real, and reported on the challenge of weighing the more present risks of vaccination against the small, future risk of cervical cancer for their children [39].

Some Indigenous people in the literature were uneasy about the possibility of infertility or sterilization. Community participants in Papua New Guinea feared that the HPV vaccine would result in the destruction of reproductive organs and trigger additional complications resulting in infertility [32]. Other studies echoed concerns about infertility arising from the vaccine [41, 45]. In communities where children and youth are prioritized, the potential for infertility is a serious health concern with important socio-cultural ramifications [40].

#### Distrust

Deep distrust of the institutions providing the HPV vaccine can be a barrier to uptake in Indigenous populations. Many communities engage in sharing stories and disseminating oral knowledge, including powerful stories of lived experiences of injurious or disabling interactions with the healthcare system. Social communication of experiences of perceived harm from vaccines and ill-intent of institutions can result in community-wide caution, distrust, and general apprehension towards HPV vaccination [49]. Information passed down through generations may reflect long standing suspicion of government or healthcare system intent in distributing vaccines to Indigenous people. In the United States, participants assumed that, and questioned why, a new vaccine was being ‘tried out’ among Native women first [39], and many questioned the intentions behind vaccine distribution to their communities, assuming that many factors were yet unknown. One health worker in Ontario, Canada spoke of stories of a vaccine circulating in previous years that was meant to kill off Indigenous communities [23]. General distrust in the government, pharmaceutical companies, and healthcare workers was evident. There was widespread concern that any ‘new’ vaccine might not only be unsafe but tested on Indigenous communities without their consent. One Alaska Native parent expressed alarm at her suspicion that Indigenous people could be treated as laboratory test animals, having heard stories of past Indigenous people being unknowingly or forcibly vaccinated [48]. Some parents in Australia expressed that the vaccine’s effectiveness could not be known because the vaccine had not been circulating long enough. One woman stated, “We’re guinea pigs! The vaccine has not been around long enough because the ‘experts’ do not know the long-term effects. …it hasn’t yet proven to be effective” [40].

Mistrust of any information stemming from pharmaceutical companies was highlighted by participants in Alaska [48]. Fear of alleged nefarious reasons for physicians and pharmaceutical companies recommending HPV vaccinations was evident. A vaccine-eligible young adult asked why American Indian women were being targeted and wondered whether the campaign was meant to decrease their population [41]. Several participants in the same study noted that fear about, and mistrust of, the federal government is persistent. One student observed that vaccinations for Indigenous people have a ‘crazy’ history that results in low trust in the health services system [41].

#### Lack of effective knowledge dissemination

In multiple studies, vaccine-eligible young adults, parents/caregivers, and health workers alike expressed a desire for increased understanding of HPV and the vaccine. Participants asked for explicit details about HPV vaccines to allay concerns, to educate their family members, and to make informed decisions about their health, the health of their children and the health of their communities [28, 40, 44].

Across the majority of studies reflecting Indigenous voices, there were low knowledge levels regarding both the HPV vaccine and the disease outcomes from HPV infection. Parents and caregivers stated that insufficient knowledge of the HPV vaccine was a barrier to providing consent for their children [40, 50], and that they would like more information [48, 51]. Parents and caregivers in a Utah-based study described “receiving basic knowledge of the HPV vaccine through their healthcare provider”, but “few had in-depth knowledge about the vaccine” [49].

Despite a pharmaceutical industry marketing campaign in Alaska prior to a study [47], vaccine-eligible youth knew little about HPV, the HPV vaccine, or cervical cancer. Misunderstandings about HPV disease outcomes and modes of HPV transmission were common among vaccine-eligible young adults attending universities in the United States [42], with one student suggesting that low knowledge levels exist in American Indians due to low educational efforts in their communities [41].

Insufficient knowledge of the HPV vaccine was also an issue for the healthcare providers serving Indigenous communities, with one study identifying misunderstandings regarding vaccine eligibility requirements and beliefs about age-appropriateness among providers in Washington State [38]. In a school-based vaccine initiative in New Zealand, over 77% of surveyed teachers and support staff expressed that they would like more information about the vaccine before they could feel comfortable discussing it with their pupils or the pupil’s parents [52].

#### Lack of culturally appropriate awareness campaigns

Loring (2008) noted that Indigenous children from families with low awareness of the HPV vaccine are more likely to be overlooked in vaccination programs, especially when HPV and cervical cancer are not talked about within communities. In survey data, Indigenous people reported that one reason they had not consented to vaccination of their son or daughter was that they did not know about the vaccine [34, 35, 41, 46]. Prior to HPV and cervical cancer campaigns in Fiji, only 10% of respondents reported having heard of HPV or the vaccine [46]. A retrospective review of registry data from New Zealand reported that the overall awareness of HPV as a risk factor for cervical cancer was lower among Maori parents than those of European descent [53]. When asked about barriers to HPV vaccine uptake, Indigenous physicians in the United States reported that lack of parental awareness is an issue for their Indigenous patients. Among healthcare providers, a lack of patient awareness about vaccine benefits was listed as one of the ‘top five barriers to HPV vaccination’ at their facilities [54].

#### Gender- and age-specific disparities in health education delivery

Some researchers reported knowledge disparities concerning gender equity in contracting HPV; in one study, parents were generally unaware that the HPV vaccine was recommended for males as well as females [49], and female participants reported greater awareness of HPV than their male counterparts [42]. Vaccine-eligible young adults were confused about whether HPV was something men could get [44], and Aboriginal parents residing in Melbourne stated that HPV vaccine promotion should be more gender-balanced [40]. Participants noted that the resources available for HPV vaccination seem biased with female-centric messaging; without considering the cultural sensitivities around educating men about the shared infection of HPV, it would continue to be seen as a ‘women’s disease’ [40]. In certain contexts, specific barriers to consent for men emerged, highlighting how the delivery of HPV health education may be gender-disparate. The need to sufficiently inform men (including single-parent fathers, male grandparents, and so on) was identified as important, especially for sole parents and caregivers who are responsible for providing immunization consent [40].

A Canadian workshop with Indigenous vaccine recipients highlighted issues with age- and culturally-inappropriate health resources for the HPV vaccine [55]. Parents in Australia felt that culturally relevant materials were important resources to create an inclusive information environment for young adolescents [40]. One element of a culturally disparate approach to HPV vaccine education in communities was the exclusion of Elders from the health education process, and the perceived lost opportunity to foster intergenerational openness. During focus group talking circles in a community gathering in Western Canada, some Elders expressed disappointment to learn that their grandchild had been vaccinated without their knowledge or support, stating that the talking circles were the first time they had heard about HPV [28].

#### Lack of healthcare provider recommendation

If their healthcare provider did not recommend the HPV vaccine or provide education, then parents and caregivers were less likely to vaccinate their children [34, 35, 44]. Interview participants from the United States cited weak or inconsistent provider recommendations as barriers to vaccination [56]. Staff turnover, lack of provider time, and recommendation inconsistencies among providers were also noted as contributing to a lack of trust and confusion that Indigenous patients might feel when faced with the decision to consent to HPV vaccination [54, 56]. As stated in a study with parents and caregivers in Utah, communication failure through lack of trust would result in parents seeking HPV vaccine information elsewhere [49].

#### Colonial systems and processes

In some studies, there were reports of minimal efforts to ensure consent was fully informed and freely given before vaccinations were administered. Parents in Papua New Guinea said that they did not know what their children’s routine immunizations (outside of HPV) had been for, leaving them unaware as to what their children were protected against, often due to poor health messaging provided by health workers at the time of immunization [57]. Additional and complex issues related to authority in providing consent for vaccination when the vaccine-eligible child lives in a foster-care system were raised in Australia, as well as questions about signing the consent form if they were an unofficial guardian, and if the child is not registered as a ‘ward of the state’ [40]. The HPV vaccine consent processes created apprehension among some Victorian communities, leaving parents expressing frustration with the complexity of the consent form, the challenging or confusing information provided, and the lack of detail on long-term safety. In contrast, despite some complications in understanding the technical information, other participants in the same community found the HPV vaccine consent form had sufficient information to inform their decision [40].

In key informant interviews in New Zealand, a Maori health expert highlighted the importance of ensuring that existing colonial systems continue to support equitable HPV vaccine uptake for the Maori people [58]. The Maori health expert noted that Maori women are especially vulnerable to health policies designed around the total population, and that these policies are inherently racist and will naturally generate and perpetuate inequities.

#### Social determinants of health service access

Even when the HPV vaccine was offered free of charge, a Peruvian study reported that the availability of medical staff, equipment, and facilities can be a barrier for access, especially in remote communities [59]. For those who missed vaccination in a school-based program, lack of health insurance, lack of public funding, and an inability to pay out of pocket prevent older Indigenous people from completing the HPV vaccine series [37, 38, 42, 60]. For Shipibo-Konibo women in Peru, poverty was cited as a barrier in accessing the HPV vaccine [59], and low socioeconomic status was associated with an incomplete HPV vaccination series in Australian reviews of large-scale vaccination programs [43, 61]. Public health and policy leaders in key informant interviews objected to a proposed policy to make the HPV vaccine privately available, citing the potential disparities with low income Maori families being the least able to afford the vaccine for their children [58].

Indigenous people in rural or remote communities face additional barriers in terms of physical access to clinics and transportation issues [42, 43]. Physical relocation [45] and poor school attendance [62] contributed to incompletion of the HPV vaccine series in Peru and Australia, respectively. For some communities in Central Australia, their remote environment created challenges that required collaboration to overcome. To encourage youth to complete the full dose schedule, the support of the entire community was warranted, including the influence of Elders and community networks [40]. The timeliness required in a multi-dose vaccine schedule and issues with patients returning for subsequent doses were also reported by the authors of retrospective reviews examining Australian immunization programs as a barrier to completion of the HPV vaccination as recommended [43, 61].

### Thematic supports for HPV vaccination

#### Beliefs

Communicating the importance of receiving the HPV vaccine through the lens of ‘cancer prevention’ might be important to achieve optimal HPV vaccine uptake. Australian parents who were also health workers thought it was important to promote the vaccine as a cancer prevention strategy [40], because the perception of future disease prevention would increase vaccine acceptability. In Alaska, one parent said that the desire to protect one’s child against cancer is a part of motherhood [48]. For families who live with the trauma of poor cancer outcomes, shielding their daughter from “one less cancer” was a compelling argument for consenting to vaccination [48]. A preference for promotional messaging focused solely on cervical cancer was also reported by a handful of men, who believed that cervical cancer prevention should be prioritized. So concerned was one community member in Papua New Guinea by what he saw as the burden of the disease in the community, he wanted the vaccine to be promoted “to help young women and even men to prevent this cancer disease” [33].

#### Affirmative multigenerational and social influences

Parents and caregivers have a role to play in communicating the importance of the HPV vaccine [48, 51, 52], and interest in vaccination reported by parents has been found to be predictive of future consent for vaccination [36]. Offering HPV vaccine education to all adult women in the community allows all women to adopt continuing roles as community educators of each other [40].

Teachers in New Zealand suggested involving youth as campaigners and role models to provide encouragement for their peers, as well as involving youth-friendly media in HPV vaccination campaigns [52]. The social influence of peer support can mitigate vaccine-related anxiety and encourage vaccine normalization and ownership. In Canada, participants described the children of their community as educators, supporting each other through the immunization process regardless of bravery or fear. The supportive environment for and by youth during the vaccination process was encouraged through community celebration and acknowledgement [28].

#### Increased HPV knowledge

Greater knowledge of HPV or the HPV vaccine was found in multiple studies to increase parents’ and caregivers’ willingness to consent to HPV vaccination [38, 41, 46, 59]. This finding was not consistent with an online survey of young female university students in Arizona; those who were unvaccinated demonstrated comparable HPV knowledge to those who were vaccinated against HPV [63].

#### Community-oriented health education delivery

Encouraging community-level discussion of the HPV vaccine and its protective effects was viewed as a viable method to support vaccination uptake in many Indigenous communities [40]. Sharing information across communities through educational support sessions was considered helpful in normalizing the HPV vaccine, taking ownership of health, and encouraging vaccine-recipient autonomy, regardless of age. Intergenerational health education, as well as the social influence of community leaders and Elders were thought to be necessary components of any vaccine education program. Participants voiced frustration that the Elder women were not able to take part in the conversations due to a lack of knowledge provided specifically to the Elders. Comments about desiring the involvement of wise female women in community HPV education were persistent [40]. Some community members said that they were willing to learn more about the vaccine, especially if the vaccine education opened doors to increased communication with their daughters and granddaughters. Once equipped with reliable health information, parents and grandparents in western Canada felt prepared to support their community’s youth, discuss issues related to sexual intercourse, and foster the community transparency believed to be protective of health [28].

It was suggested that educational efforts must discuss not only the protection afforded by the HPV vaccine, but also frank information about possible adverse effects. Young people, in particular, expressed the need in Papua New Guinea for information on the vaccine’s advantages, disadvantages, and side effects [33]. Parents and caregivers in Australia also thought that, despite what information was included, health education content must be rooted in truth and integrity, and presented at a language level compatible with the adolescent’s age [40]. Increasing a community’s overall knowledge of the vaccine and of the disease, when done in ways that resonate for the community, can motivate hesitant parents to make decisions in favour of the HPV vaccine. A mother in Peru stated that her initial worry over the early age of the vaccine was replaced with a feeling of happiness after she received proper explanation on the topic [59]. Once participants had been provided with transparent, factual information, their attitude toward the HPV vaccine often changed and they felt confident in discussing the HPV vaccine with their child and as a result, provide truly informed consent [40].

For parents in Victoria, Australia, community education was considered vital in the consent process. This education was also critical in unwinding any sexual implications that community members have attached to the HPV vaccine [40]. Equitable community education conducted with cultural and gendered needs in mind might be valuable in overcoming the HPV vaccine barriers associated with sex and cultural taboos. While some mothers in Alaska reported that the decision to consent to vaccinate their children was their decision alone, for some families it was a shared responsibility between partners [48], reflecting that men cannot be excluded from HPV vaccine education. To build consistent community understanding, female focus group participants in Papua New Guinea wanted boys and men to better understand their role in HPV transmission and the risks borne by females and males of cancer caused by the disease [33]. In a study of semi-structured interviews with parents in Peru, the basic needs in terms of health education delivery were outlined as such: provide good information from a trustworthy source, and to both parents [59].

#### Consistent healthcare provider recommendation

Healthcare providers making a strong recommendation for HPV vaccination is seen to support Indigenous people in receiving the vaccine [35, 37]. Information received from a healthcare provider is generally trusted provided that there are positive relationships between the healthcare provider and their patients in the community [36, 39, 49, 62]. For healthcare providers serving Indigenous populations in Michigan, best practice to promote the HPV vaccine is to review immunizations at every patient visit, remind them of their eligibility and make strong recommendations for the vaccine [56]. Members of the Association of American Indian Physicians affirmed their willingness to provide HPV vaccinations to both female (98%) and male recipients (88%) [60].

#### Equity-oriented systems and processes

Directing resources to create a position for a dedicated immunization nurse [37], or further supporting nurses immunizing in schools [58] are strategies that could help alleviate immunization inequities for Indigenous populations. Out-of-the-box thinking such as ‘door knocking’ to obtain the consent of a parent or caregiver [62], or obtaining verbal parental consent via telephone [37] have been proposed as unique strategies to increase HPV vaccine delivery. Community members from Papua New Guinea expressed the importance of any HPV vaccination campaign timing and the preparatory work required to ensure communities are informed and ready to receive a vaccine prior to its widespread delivery administration [32].

Reminders and follow-up from healthcare providers might be helpful in encouraging parents to complete the consent form for HPV vaccination [43]. The majority of Maori parents in New Zealand said that they would be happy to receive a telephone call reminder, had they not returned the consent form [51]. Communications strategies that address local issues affecting HPV vaccination, including education that increases both provider and patient knowledge about the HPV vaccine, are systems-level changes that have been proposed by public health leaders and healthcare providers alike [54, 58]. Collaboration with the community can mitigate many barriers [62]. To address issues of cultural safety, community leaders should be involved in planning health promotion activities to ensure they are effective [55].

#### Social and cultural determinants of health

Physical access to the immunization clinic (or school) supported the receipt of HPV vaccination [62], as did having a high relative socioeconomic advantage [43]. In Australia, interview participants were supportive of the government subsidization of the HPV vaccine because it removes the barrier of cost. One participant stated that a free vaccine would increase the number of girls getting vaccinated [40].

Initiation of the vaccine series at a younger age has been a successful strategy to increase uptake in Indigenous populations. In the United States, Indigenous children who initiated the HPV vaccine series at age 9 or 10 were 2.5 times more likely to complete the series compared to those who started at the recommended age of 11 or 12 [56]. The authors suggest that children who initiate the HPV vaccine series before the recommended age had parents who were more proactive about preventive care, and that clinical implications could be important as far as the timing for providers to communicate about HPV vaccine with parents. Meanwhile, Indigenous parents in New Zealand were significantly more likely than their other ethnicity counterparts to select younger ages to be appropriate for vaccination [51]. It has also been suggested that starting HPV vaccination earlier than the recommended age could result in higher dose completion rates by giving Indigenous children a head start [37].

For Indigenous community members in the United States, participation in a traditional cultural practice was found to be significant. Those who participated in ceremony were two and a half times more likely to receive the HPV vaccine [64].

In one study in Fiji, the majority of parent respondents included their daughter in the decision-making process [46], and in Alaskan focus groups, mother-identifying parents reported they involved their older teenage daughters, as well as their spouses, in the decision about vaccination [48]. Parents in New Zealand reported that 34% would seek the views of their daughters, 42% would seek the views of friends/peers, and 50% would seek the views of their extended family in the decision to vaccinate their child [51]. Involving the whole of the community in the decision and providing a degree of autonomy to the vaccine recipient themselves might mitigate barriers such as distrust and lack of knowledge [40]. Although there were opinions about the complex nature of cancer prevention and sexual health messaging, some participants in Australia voiced the need for HPV vaccine resources to be communicated specifically to young girls, suggesting that early education would encourage widespread acceptance of the HPV vaccine [40].

## Discussion

The primary aim of this systematic review was to identify barriers to and supports for HPV vaccination for global Indigenous people, as reported in the literature. In the results section, we have presented the findings as reported by both Indigenous and non-Indigenous study participants. In this discussion, we focus on our secondary aim, namely reflection on the barriers and supports specifically reported by Indigenous participants, including Indigenous parents/caregivers, health workers, community members, and vaccine-eligible youth. Our research team, which includes our First Nations research partners, offer this discussion to provide unique insight and give voice to Indigenous perspectives wherever possible. Given that the majority of our team (Indigenous and non-Indigenous) are based in Canada, we have included some additional reflections specific to the Canadian context in S2 Text, which may be of interest to Canadian readers.

### Indigenous parent and multigenerational caregiver perspectives

The studies included in this review illustrate the hesitancy of Indigenous parents and caregivers to vaccinate their children against HPV due to concerns about sexual health, uncertainty about vaccine safety, and general distrust of colonial systems. While HPV vaccination is not associated with an increase in sexual activity [6, 65, 66], parents and caregivers acknowledged the complexities of overcoming the social stigma associated with sex and cultural taboos. While not explored in the literature reviewed, this specific form of vaccine resistance could be a result of a multi-generational legacy of colonization, including the influence of church indoctrination on middle and older generations in Indigenous communities. Questioning the role and effects of assimilationist, oppressive colonial systems on the beliefs and behaviors of Indigenous peoples globally may provide future researchers with additional understanding beyond surface level barriers related to sexual taboo.

Fear of sterilization or other feared vaccine injury was driven by lived or intergenerational experience with harmful practices whereby Indigenous people were used as nonconsensual research subjects [67]. Members of Indigenous communities might be wary of the vaccination process due to historical and lived experiences with medical or government systems; this legacy of racist paternalism cannot be ignored in understanding vaccine hesitancy for Indigenous people [68]. Despite their hesitations, parents and caregivers expressed the desire for increased understanding of the HPV vaccine, and were supportive of messaging pertaining to the vaccine’s cancer prevention benefits. In this light, the process of informed consent, providing in-depth knowledge about short and long-term vaccine safety details, and education of the entire community are a vital practice. It should be emphasized that upon receiving adequate HPV vaccine education, many Indigenous parents were open to earlier ages of vaccine initiation and approached the vaccination process in a holistic, intentional way (involving extended family members, the vaccine-eligible child, and the wider community in a process of support).

### Indigenous healthcare worker perspectives

Inadequate awareness of community vaccination needs can contribute to barriers in vaccine service provision for Indigenous people [69]. Although Indigenous healthcare workers reported feeling insufficiently informed to be the sole HPV vaccine educators of their community, they did seek knowledge for themselves; support from the media for vaccine messaging, especially in remote communities, might alleviate some of this pressure. Healthcare workers cited parental distrust of vaccination due to concerns related to sexual behavior and fears about vaccine safety. They also noted that HPV vaccination seems to be a low priority for members of their community.

The ability to address health issues within Indigenous communities is often dependent upon the capacity of the healthcare workers themselves. Responding to health crises can overshadow other concerns despite a recognized need to address them. In these instances, health researchers can play a key role by providing educational resources to healthcare workers to build capacity in the community and to ensure meaningful actions are taken that reflect a deep level of commitment to supporting the community. One example of such support can be found in actions that are taken to demonstrate a true sense of reciprocity. Reciprocity was exemplified by a recent series of Métis-led vaccination clinics against SARS-CoV-2 in Alberta, Canada, during the COVID-19 pandemic. The inclusion and broad support of Indigenous health workers by the provincial health authority, non-Indigenous healthcare workers, and leading researchers in the study resulted in a low-barrier, culturally safe immunization space for presenting vaccine recipients [70]. Other examples of support might include assisting the staff with challenges they face related to the work at hand, such as providing in-service educational sessions, collecting data that aligns with the research interests of the community, or assisting program staff to make positive changes in health programming at the community level, wherever appropriate.

### Indigenous community member perspectives

Community members expressed disappointment in the colonized approaches to HPV vaccine health education, acknowledging that the exclusion of Elders and the use of culturally inappropriate resources might contribute to lower vaccination rates for their communities. Enabling HPV vaccine health education for all community members was seen as a more culturally traditional way of sharing knowledge, with intergenerational health education working to normalize the HPV vaccine. Prior to colonization, Elders might have been traditionally involved in cross-generational health education, and with the right support, it is possible for Elders to reclaim these roles and to guide young people in decision-making that protects health [71]. In providing consent, a wider generalized community consent process (beyond the immediate parent or caregiver) might be a more culturally appropriate approach that is protective of youth, while allowing for some youth autonomy around their own health decision making.

### Indigenous vaccine-eligible young adult and youth perspectives

For vaccine-eligible young adults over the age of 18, fears of vaccine injury and concerns about Indigenous women being ‘targeted’ and harmed by vaccination effectively limits HPV vaccine acceptance. The combination of a low-risk perception of HPV and a perceived risk avoidance through cancer screening presents difficulty in shifting the risk-reward beliefs of these young adults. The assessment lens is an example of something education can affect by encouraging young people to recognize the long incubation periods of HPV-related cancers and to understand the limitations inherent to screening or healthcare requiring detection to occur early.

Only six studies investigated the perspectives of Indigenous young adults; more research on this topic is required. A lack of youth participation in the literature represents a clear and persistent obstacle to HPV vaccination strategy development. Knowledge translation strategies might not be effective if there is little understanding of the needs of the vaccine recipients. These factors have negative implications for both HPV vaccine strategy development and for HPV-related disease prevention. Research conducted by young adults with young adult participants might yield candid responses that could provide a helpful basis for such strategy development.

Only three studies highlighted the voices of Indigenous youth under the age of 18 years. Despite this fact, we have made every attempt to highlight youth concerns because evidence suggests that tailoring vaccine strategies to the needs and preferences of the target adolescent population might increase uptake [72]. Indigenous youth under 18 years of age reported that community-held beliefs about sex, concerns about vaccine injection pain and side effects, and their own lack of knowledge acted as barriers to HPV vaccination. Youth explained that transparent HPV vaccine education offered to all genders would help increase community knowledge, positively influencing the social support of parents in providing consent for the vaccine. The published literature contains calls to understand the barriers and supports that exist for Indigenous youth themselves to tailor HPV vaccination intervention strategies to meet youth needs [13, 73].

The perspectives of Indigenous youth for a service that is directed at this population are paramount given the barriers and supports identified. For example, support for HPV vaccine uptake included youth influence on parental consent for vaccination, as well as the role of youth in areas of social influence and decision-making. This finding mirrors vaccination preferences in non-Indigenous youth, where adolescents expressed the desire to be involved in the decision-making process more explicitly [74]. Further, many Indigenous people hold beliefs about the concept of non-interference, objecting to encroachment upon the rights, privileges or activities of another [75]. That is to say, decisions made for individuals against their will or without their knowledge might not be considered appropriate in a traditional sense; this belief suggests a need for increased awareness of HPV and the HPV vaccine among youth.

### Gaps in the literature

Over 95% of the studies we identified in this review originated from North America and Oceania, where Indigenous people are more explicitly defined and maintain shared histories of colonization. This fact is notable, given the documented global existence of Indigenous people in at least 90 nations worldwide [16]. This limited geographic representation is notable also because there is a persistent, well-documented, and disproportionate burden of HPV-related disease in Indigenous communities globally. Nevertheless, not all countries have implemented HPV vaccine programs; as of 2019, only 30% of the target global population are reached through national HPV vaccine schedules [76]. Without HPV vaccines being available to all Indigenous people, the burdens of HPV infections will persist.

We also noted a general lack of embedded Indigenous consultation and involvement in the studies included in this review. This is concerning, given the well-documented importance of early and consistent community participation in improving the acceptability, applicability and success of Indigenous health research. Also, due to the challenges associated with recruiting minor-aged children, children who are eligible for HPV vaccination in many countries (e.g. through school-based HPV vaccination at ages 9–10 years, in Canada) have been excluded from participation in most research studies.

Issues of racism and discrimination experienced by Indigenous people in accessing healthcare were limited in the findings of this systematic review. Given that the majority of the studies included did not situate the identity of the researchers (i.e. their Indigenous or non-Indigenous identities), we do not know if the authors of the studies included in this review approached their work with a lens geared towards identifying the multifaceted effects of colonization. This lack of deep engagement with Indigenous people’s experience of racism is an important gap in the current knowledge base which should be explored in future studies.

More subtly, the legacy of systematic exclusion of Indigenous voices from healthcare decisions indirectly points to issues of racism as a barrier to HPV vaccine uptake for Indigenous communities globally. The barrier of deep distrust towards the institutions (government, health care, and pharmaceutical companies) recommending and providing the HPV vaccine is one example of intergenerational trauma caused by colonizing systems and oppressive practices, many of which continue today. Likewise, the lack of consistent, culturally-appropriate engagement by researchers is evidence of embedded colonizing practices at work. Although these issues were indirectly addressed in the literature, it is important to highlight that racism and discrimination prevent the full participation of Indigenous people in the health care system, and the insidious nature of structural racism—often subliminal and hard to identify—is a recognized barrier to health care access [77]. It is essential to understand the role and impact of racism on HPV vaccine uptake to ensure that resources are created to address the harmful effects of distrust caused by systemic racism.

The COVID-19 pandemic has further highlighted the need for adequate data and information on the health of Indigenous populations [78, 79]. Further evidence-based improvements across health systems are needed, including with regard to the growing cancer care needs of Indigenous people globally.

### Limitations and strengths

There are a number of noteworthy limitations to our study. We experienced difficulty in identifying Indigenous populations within the literature and in how studies reported on the populations due to the varying ways these populations are defined [16]. In addition, the QATSDD tool we used to assess study quality has become outdated since we conducted our analysis [30]; a refined version has recently been created in response to the limitations of the original tool [80]. There also exists a separate assessment tool that appraises research quality from an Indigenous perspective [81]; we would have chosen this method over the Western-perspective tool that was used had it been available at the time.

In presenting the barriers and supports for HPV vaccination separately for Indigenous and non-Indigenous people in the literature (Table 2), we note that we have not engaged in in-depth analysis of study design quality. We were limited to the information available in the published work and did not have access to the tools used to collect data for the primary research. Having access to this information may have helped us to understand why racism was not identified as a major barrier to immunization. Any inadvertent bias as a result of this presentation might limit the reader’s understanding of the relationship between study design and the barriers or supports reported by Indigenous people.

We acknowledge that each community of people defined as ‘Indigenous’ is distinct, and the needs regarding HPV vaccine acceptance will vary due to cultural contexts situated within unique historical and current experiences of colonization. We acknowledge that the perspectives offered by our First Nations team members are not reflective of all Indigenous people within Canada or globally. Alternative interpretations might emerge if additional Indigenous voices were included in our team’s work.

While there is no ‘one-size fits all’ solution to increasing HPV vaccine uptake among Indigenous people, we have systematically identified the extant barriers and supports to HPV vaccination for global Indigenous people as reported in the peer-reviewed and grey literature. Our focus on the Indigenous voices present in the literature highlights and distinguishes Indigenous perspectives from those voices of a Western background, and this work is critical to advancing HPV vaccine uptake. From its inception, our study team has been comprised of both Indigenous and non-Indigenous collaborative members; key understandings of the results presented here were co-led and co-interpreted. It is our hope that future HPV vaccine research involving Indigenous participants will center Indigenous voices and reflections in scholarly discussion and dialogue.

The methodology of this research sought to bridge Indigenous ‘Ways of Knowing’ with Western scientific research processes. Although the methodology can refer to principles of community-based research for the benefit of enabling a holistic perspective of the project findings and outcomes, as previously noted, the collection, analysis and synthesis of outcomes relies on Indigenous interpretations and meanings to ensure the voices and perspectives of First Nations are given prominence in the findings [82, 83]. Therefore, the analysis of systematic review outcomes utilized a “collaborative approach” that equitably involved all partners and recognized the unique strengths that each brings to the process.

### Recommendations

The perspectives of Indigenous vaccine-eligible children, youth, young adults, and Elders are underrepresented in the literature, creating a knowledge gap in understanding the barriers and supports to HPV in vaccination for Indigenous communities. Lack of formal investigation of child populations (due to challenges in involving them in research studies) should not preclude consideration of their input on HPV vaccine programs. The insights and preferences of this population should be captured and considered when developing and delivering local HPV vaccination programs within Indigenous communities. To appropriately inform vaccination policy worldwide, increased understanding of the perspectives of vaccine-eligible Indigenous children, youth and young adults should be prioritized, along with the perspectives of community Elders. In addition to exploring youth perspectives through an academic lens, potential approaches would be to involve Elders, parents, and grandparents (with special consideration taken to include male-gendered individuals) in collective efforts to inform communities about HPV-related cancers, HPV vaccination, and sexual health education. In many Indigenous cultures, Elders hold positions of influence and respect in communities as knowledge-holders and community guides [84, 85], and might be ideally situated to lead collective discussion about the cancer preventing benefits of HPV vaccination [28].

Based on findings from the literature, coupled with interpretation and dialogue from Indigenous team members, we have compiled the following general recommendations regarding HPV vaccination in Indigenous populations. We propose that it is essential for researchers, healthcare providers and policy-makers to:

1. Prioritize collaboration with Indigenous leaders, Elders, children and youth.
2. Ensure HPV vaccination is offered through multiple venues and various locations, allowing individuals to access HPV vaccines both within and outside of Indigenous communities.
3. Focus on vaccine relevance, normalization, and ownership, and ensure Indigenous representation in media messaging (with a focus on oral traditions and story sharing).
4. Address misconceptions about HPV vaccines and HPV disease risk perception through community-level discussion and educational support sessions.
5. Foster intergenerational openness in community health education, with a focus on engaging Elders in knowledge gathering.
6. Engage children and youth to receive their comments on what youth need regarding HPV education and knowledge to make informed familial decisions about the vaccine.
7. Involve the men of the community; their knowledge and participation are an important component for communities when discussing sexual health promotion.
8. Be honest and maintain full transparency at each stage of the knowledge-sharing process, working at the pace of the community, and ensuring fully informed consent prior to HPV vaccination.

## Conclusion

To enhance HPV vaccination for Indigenous populations globally, culturally-appropriate, collaborative, and inclusive health education for all community members might assist governments and vaccine providers to normalize the HPV vaccine, to address misconceptions about the HPV vaccine and to ensure fully informed consent. Future work to increase HPV vaccine uptake should be supportive of community-based research in partnership with Indigenous people, to ensure the richness of shared knowledge and to reflect accurately the holistic experiences of the young HPV vaccine recipients as well as their wider community.

## Supporting information

S1 Text MEDLINE search strategy. (DOCX)

S2 Text HPV vaccination among Indigenous populations in the Canadian context. (DOCX)
