# Supplementary material for: Barriers and supports for uptake of human papillomavirus vaccination in Indigenous people globally: A systematic review
Source: PLOS Glob Public Health. 2023 Jan 6;3(1):e0001406. doi: 10.1371/journal.pgph.0001406 (PMC10021254; doi:10.1371/journal.pgph.0001406)
Supplement: S1 Text — (DOCX) [file pgph.0001406.s001.docx]

**S1 Text**

**MEDLINE search strategy**

Controlled vocabulary and keyword terms related to immunization or HPV were combined with terms related to Indigenous populations. Filters designed to retrieve studies on Indigenous populations in Canada were used in the MEDLINE, Embase, and CINAHL databases (Campbell, Dorgan & Tjosvold, 2021), as well as terminology related to U.S. and global Indigenous populations. The search strategy was developed in MEDLINE, then translated to the syntax of the other databases.

1. exp Immunization/ or exp Immunization Programs/ or exp Vaccination/ or exp Vaccination Refusal/

2. exp Papillomavirus Infections/ or exp Genital Neoplasms, Female/

3. (hpv or human papilloma* or vaccin* or immuniz* or immunis* or revaccinat*).kf,tw.

4. 1 or 2 or 3

5. exp american native continental ancestry group/ or exp oceanic ancestry group/

6. exp Health Services, Indigenous/

7. exp United States Indian Health Service/

8. (aborigin* or Alaska* native* or Aleut* or American Indian* or Amerindian* or Eskimo* or community-based participatory or Fiji* or indigenous or Inuit* or Maori or Maya* or Metis or Native American* or Native Hawaiian* or native people* or Papua* or Polynesia* or Sami or Samoa* or tribe* or tribal or Pacific Islander* or Torres Strait Islander*).kf,tw.

9. 5 or 6 or 7 or 8

10. (exp Indians, North American/ or exp Inuits/ or exp Health Services, Indigenous/ or exp Ethnopharmacology/ or Athapaskan.mp. or Saulteaux.mp. or Wakashan.mp. or Cree.mp. or Dene.mp. or Inuit.mp. or Inuk.mp. or Inuvialuit*.mp. or Haida.mp. or Ktunaxa.mp. or Tsimshian.mp. or Gitsxan.mp. or Nisga'a.mp. or Haisla.mp. or Heiltsuk.mp. or Oweenkeno.mp. or Kwakwaka'wakw.mp. or Nuu chah nulth.mp. or Tsilhqot'in.mp. or Dakelh.mp. or Wet'suwet'en.mp. or Sekani.mp. or Dunne-za.mp. or Dene.mp. or Tahltan.mp. or Kaska.mp. or Tagish.mp. or Tutchone.mp. or Nuxalk.mp. or Salish.mp. or Stl'atlimc.mp. or Nlaka'pamux.mp. or Okanagan.mp. or Sec wepmc.mp. or Tlingit.mp. or Anishinaabe.mp. or Blackfoot.mp. or Nakoda.mp. or Tasttine.mp. or Tsuu T'inia.mp. or Gwich'in.mp. or Han.mp. or Tagish.mp. or Tutchone.mp. or Algonquin.mp. or Nipissing.mp. or Ojibwa.mp. or Potawatomi.mp. or Innu.mp. or Maliseet.mp. or Mi'kmaq.mp. or Micmac.mp. or Passamaquoddy.mp. or Haudenosaunee.mp. or Cayuga.mp. or Mohawk.mp. or Oneida.mp. or Onodaga.mp. or Seneca.mp. or Tuscarora.mp. or Wyandot.mp. or Aboriginal*.mp. or Indigenous*.mp. or Metis.mp. or red road.mp. or "on reserve".mp. or off-reserve.mp. or First Nation.mp. or First Nations.mp. or Amerindian.mp. or (urban adj3 (Indian* or Native* or Aboriginal*)).mp. or ethnomedicine.mp. or country food*.mp. or residential school*.mp. or ((exp Medicine, Traditional/ or traditional medicine*.mp.) not Chinese.mp.) or exp Shamanism/ or shaman*.mp. or traditional heal*.mp. or traditional food*.mp. or medicine man.mp. or medicine woman.mp. or autochtone*.mp. or (Native* adj1 (man or men or women or woman or boy* or girl* or adolescent* or youth or youths or person* or adult or people* or Indian* or Nation or tribe* or tribal or band or bands)).mp.) and (exp Canada/ or (Canad* or British Columbia or Columbie Britannique or Alberta or Saskatchewan or Manitoba or Ontario or Quebec or Nova Scotia or New Brunswick or Newfoundland or Labrador or Prince Edward Island or Yukon Territory or NWT or Northwest Territories or Nunavut or Nunavik or Nunatsiavut or NunatuKavut)).mp.

11. 4 and 9

12. 4 and 10

13. 11 or 12
