# Supplementary material for: Barriers and supports for uptake of human papillomavirus vaccination in Indigenous people globally: A systematic review
Source: PLOS Glob Public Health. 2023 Jan 6;3(1):e0001406. doi: 10.1371/journal.pgph.0001406 (PMC10021254; doi:10.1371/journal.pgph.0001406)
Supplement: S2 Text — (DOCX) [file pgph.0001406.s002.docx]

**S2 Text**

**HPV vaccination among Indigenous populations in the Canadian context**

Our research team is based mainly in Canada, and includes members who identify as First Nations (Canada’s largest Indigenous group). As such, we were particularly interested in the findings as they relate to the Canadian context. Of the studies identified in this review, only four were Canadian (from the provinces/territories of Alberta, Ontario, Québec, and the Northwest Territories), which highlights a significant gap in knowledge about the perspectives of these specific populations despite the well-documented inequities in healthcare access, cancer prevention, disease burden, and subsequent treatment for Indigenous people in Canada.(1-4) Canadian studies with Indigenous participants identified in this systematic review cited issues with the HPV vaccine consent process, health services access, and a general lack of support extended to wider members of their communities beyond the parent or caregiver, contributing to the low understanding of cultural context by the vaccine ‘deliverers’. Elders and grandparents were specifically mentioned as not having a role in vaccine education and the consent processes. An important lesson derived from how HPV vaccine uptake among Indigenous people is supported in the Canadian context is that health institutions must actively engage with and respectfully listen to Indigenous leadership in order to ensure health promotion/disease prevention initiatives are effective as well as to make culturally safe spaces for those who engage in the Western-colonial healthcare systems.

*The Truth and Reconciliation Calls to Action in Canada*

The UN Declaration is central to efforts to address Canada’s legacy of colonialism, highlighted by the Truth and Reconciliation Commission as “the framework for reconciliation”.(4) A Canadian federal statute, the *United Nations Declaration on the Rights of Indigenous Peoples Act, SC 2021, C14,* reinforces the responsibilities of governments to respond to the needs of Indigenous people across Canada. Further, the *2015 Truth and Reconciliation Commission*, Calls to Action 18-24 address specifically the health of Indigenous people wherein #18 states:

We call upon the federal, provincial, territorial, and Aboriginal governments…to recognize and implement the health-care rights of Aboriginal people as identified in international law, constitutional law, and under the Treaties.

These and other treaties, statutes, and statements highlight the importance of health research with Indigenous people and the potential to make meaningful contributions toward “implementing the health-care rights” of these populations.

*Localized knowledge translation strategies in Canada*

Findings from this research study might be useful in informing the development of an Integrated Knowledge Translation (iKT) strategy to support informed decision-making and increase uptake of the HPV vaccine among First Nations communities in Alberta. The iKT approach includes engagement of Indigenous communities about HPV prevention and screening in a culturally appropriate and safe manner. Community gatherings with key informants can help to provide a basis to understanding how community practices can support the health promotion and illness prevention needs of these communities in relation to the HPV vaccine barriers and supports. More specifically, Indigenous perspectives on HPV education, information, and vaccine uptake might then be used as a foundation to create a framework that better addresses the needs and priorities of these communities, and to contribute to the design and application of a KT model and approach for Indigenous communities. Continuing relationship building and engagement during this project demonstrates the importance of working in partnership with Indigenous communities to produce outcomes reflective of their needs, now and into the future.

**References**

1. Hamlin-Douglas, L. K., Coutlee, F., Roger, M., Franco, E.L., & Brassard, P. (2008). Prevalence and age distribution of human papillomavirus infection in a population of Inuit women in Nunavik, Quebec. Cancer Epidemiol Biomarkers Prev,17(11):3141–9.
2. Jiang, Y., Brassard, P., Severini, A., Mao, Y., Li, Y. A., Laroche, J., Chatwood, S., Corriveau, A., Kandola, K., Hanley, B., Sobol, I., Ar-Rushdi, M., Johnson, G., Lo J., Ratnam, S., Wong, T., Demers, A., Jayaraman, G., Totten, S., & Morrison, H. (2013). The prevalence of human papillomavirus and its impact on cervical dysplasia in northern Canada. Infect Agents Cancer, 8(25):1–11.
3. Louchini, R., & Beaupré, M. (2008). Cancer incidence and mortality among Aboriginal people living on reserves and northern villages in Quebec, 1988–2004. International Journal of Circumpolar Health, 67(5), 445-451.
4. Young, T. K., Kliewer, E., Blanchard, J., & Mayer, T. (2000). Monitoring disease burden and preventive behavior with data linkage: cervical cancer among aboriginal people in Manitoba, Canada. Am. J. Public Health, 90(9), 1466–1468. https://doi.org/10.2105/ajph.90.9.1466
5. Truth and Reconciliation Commission of Canada. (2015). *Truth and Reconciliation Commission of Canada: Calls to action.* Retrieved from https://ehprnh2mwo3.exactdn.com/wp-content/uploads/2021/01/Calls_to_Action_English2.pdf
